# Reply to: Longer treatment time and lower radiation doses—an alternative for Graves’ ophthalmopathy treatment

**DOI:** 10.1007/s00066-022-01937-y

**Published:** 2022-04-25

**Authors:** Thomas Weissmann, Florian Putz, Benjamin Frey

**Affiliations:** Department of Radiotherapy, Universitätsklinikum Erlangen, Friedrich-Alexander-Universität Erlangen-Nürnberg, Universitaetsstr. 27, 91054 Erlangen, Germany

We thank Dr. Onal and Dr. Guler for their thoughtful comments and the important points raised [1]. As pointed out by Dr. Onal and Dr. Guler, it was indeed an important observation that the rate of adverse effects was higher in the low-dose than in the high-dose cohort. An observation that requires further explanation. We have therefore explored potential reasons that explain this unexpected finding. As proposed in the manuscript, the significantly increased frequency of second radiotherapy courses in the low-dose group is one particularly plausible reason that might explain this observation in part or in full. Importantly, in a post-hoc analysis, when excluding the patients who were treated with a second course of radiotherapy, the difference in reported side effects between the low- and high-dose arm lost statistical significance (*p* = 0.215). Thus, we have presented a possible and plausible reason for the increased rate of adverse effects [2]. Nevertheless, we cannot fully exclude further radiobiologic, methodologic, or unknown factors, which cannot be proven by our data. We therefore thank Dr. Onal and Dr. Guler for providing an additional point of view in regard to the impact of treatment time. Indeed, a more protracted application of radiotherapy for Graves’ orbitopathy in comparison to the daily treatment scheme performed in our study may further improve functional outcomes and side effects [3–5]. However, this should not have affected the observed differences between the high- and low-dose arms in our study, since treatment was delivered daily with weekend breaks in both treatment groups. As our colleagues point out, no consensus on the optimal dose and fractionation has yet been reached in Graves’ orbitopathy. In our opinion, our study based on long-term telephone consultation of 127 patients with a median follow-up of 9.0 years provides an additional valuable datapoint for improving our collective knowledge on optimal dose and fractionation in Graves’s orbitopathy. This may include the unexpected observation of increased side effects in the low-dose arm, for which we provide an explanation in the form of increased second treatment courses, but which might only be definitively clarified by future studies on radiotherapy in Graves’ orbitopathy. While a prospective randomized design of course provides the best level of evidence and largely excludes potential biases, this has rarely been feasible in Graves’ orbitopathy [5, 6], especially not for such long follow-up periods, as presented in our trial [2]. Therefore, other study designs have to be pursued to address the important areas of uncertainty. We conducted this study to improve our understanding on the long-term comparative efficacy and tolerability of low- and high-dose radiotherapy because of a change in the fractionation scheme at our institution. The approach undertaken in the study to conduct telephone consultations provided very long-term results (median follow-up period 9.0 years), which is particularly important in a benign disease to demonstrate long-term safety combined with a long-term acceptance by the patients. In addition, this enabled the inclusion of a large number of patients. The potential for a recall bias as an additional factor influencing the increased rate of side effects with the low-dose scheme, which had been introduced more recently, was also discussed in our article, but is less likely to have played a relevant role given the findings in relation to second treatment courses. We also thank Dr. Onal and Dr. Guler for reaching out for clarification in regard to the one patient in the high-dose group who received a second series of radiation. In the “Methods” section, the general treatment guidelines in our department were described, whereas the actual treatment delivered is presented in the “Results” section. The one patient who received a second course of radiotherapy was an exception to our general treatment guideline not to deliver second radiotherapy courses in the high-dose group. We thank Dr. Onal and Dr. Guler for their valuable comments. We hope that future studies will be undertaken in our field that will shed additional light on optimal dose and fractionation in Graves’ orbitopathy, as well as on the promising benefit of protracted dose delivery.

## References

[1] Onal C, Guler OC (2021). Longer treatment time and lower radiation doses-an alternative for Graves’ ophthalmopathy treatment. Strahlenther Onkol.

[2] Weissmann T, Lettmaier S, Donaubauer AJ, Bert C, Schmidt M, Kruse F, Ott O, Hecht M, Fietkau R, Frey B, Putz F (2021). Low- vs. high-dose radiotherapy in Graves’ ophthalmopathy: a retrospective comparison of long-term results. Strahlenther Onkol.

[3] Cardoso CC, Giordani AJ, Wolosker AM, Souhami L, Manso PG, Dias RS, Segreto HR, Segreto RA (2012). Protracted hypofractionated radiotherapy for Graves’ ophthalmopathy: a pilot study of clinical and radiologic response. Int J Radiat Oncol Biol Phys.

[4] Guler OC, Onal C, Arslan G (2016). Graves’ ophthalmopathy treated with protracted hypofractionated low-dose adaptive radiotherapy: case report and review of the literature. Wien Klin Wochenschr.

[5] Kahaly GJ, Rösler HP, Pitz S, Hommel G (2000). Low- versus high-dose radiotherapy for Graves’ ophthalmopathy: a randomized, single blind trial. J Clin Endocrinol Metab.

[6] Prummel MF, Mourits MP, Blank L, Berghout A, Koornneef L, Wiersinga WM (1993). Randomized double-blind trial of prednisone versus radiotherapy in Graves’ ophthalmopathy. Lancet.

